# Immune Responses in the Glaucomatous Retina: Regulation and Dynamics

**DOI:** 10.3390/cells10081973

**Published:** 2021-08-03

**Authors:** Valery I. Shestopalov, Markus Spurlock, Oliver W. Gramlich, Markus H. Kuehn

**Affiliations:** 1 Department of Ophthalmology, Miller School of Medicine, University of Miami, Miami, FL 33101, USA; VShestopalov@med.miami.edu; 2 Department of Cell and Developmental Biology, Miller School of Medicine, University of Miami, Miami, FL 33101, USA; mspurlock@med.miami.edu; 3 Graduate Program in Neuroscience, Miller School of Medicine, University of Miami, Miami, FL 33101, USA; 4 Kharkevich Institute for Information Transmission Problems, RAS, 127051 Moscow, Russia; 5 Department of Veterans Affairs, Center for the Prevention and Treatment of Visual Loss, Iowa City, IA 52246, USA; oliver-gramlich@uiowa.edu; 6 Department of Ophthalmology and Visual Sciences, University of Iowa, Iowa City, IA 52242, USA; 7 Department of Neuroscience and Pharmacology, University of Iowa, Iowa City, IA 52242, USA; 8 Interdisciplinary Graduate Program in Genetics, University of Iowa, Iowa City, IA 52242, USA

**Keywords:** glaucoma, innate immune response, dysfunction, inflammasome, adaptive immune response

## Abstract

Glaucoma is a multifactorial disease resulting in progressive vision loss due to retinal ganglion cell (RGC) dysfunction and death. Early events in the pathobiology of the disease include oxidative, metabolic, or mechanical stress that acts upon RGC, causing these to rapidly release danger signals, including extracellular ATP, resulting in micro- and macroglial activation and neuroinflammation. Danger signaling also leads to the formation of inflammasomes in the retina that enable maturation of proinflammatory cytokines such IL-1β and IL-18. Chronic neuroinflammation can have directly damaging effects on RGC, but it also creates a proinflammatory environment and compromises the immune privilege of the retina. In particular, continuous synthesis of proinflammatory mediators such as TNFα, IL-1β, and anaphylatoxins weakens the blood–retina barrier and recruits or activates T-cells. Recent data have demonstrated that adaptive immune responses strongly exacerbate RGC loss in animal models of the disease as T-cells appear to target heat shock proteins displayed on the surface of stressed RGC to cause their apoptotic death. It is possible that dysregulation of these immune responses contributes to the continued loss of RGC in some patients.

## 1. Introduction

Glaucoma is a progressive optic neuropathy leading to dysfunction and selective loss of retinal ganglion cells (RGCs) and is the underlying cause of blindness in 80 million people [1,2]. It is a complex multifactorial disease, where elevated intraocular pressure (IOP) and aging are the major risk factors. Mechanical damage to optic nerve axons, oxidative stress, and hypoxia were shown to contribute to the pathogenesis of glaucoma by inducing mitochondrial dysfunction in RGCs, glial activation, and neuroinflammation. Chronic IOP elevation is strongly associated with the development of glaucoma, but nocturnal and spiking IOP elevation has also been linked to glaucomatous degeneration in humans [3,4] and animals [5,6]. Glaucoma-like optic nerve and ganglion cell pathology can be also induced by an abnormal translaminar pressure owing to the dysregulation of systemic blood pressure or intracranial pressure [7,8] in both humans and animals [9]. These latter mechanisms suggest that a certain proportion of “normal tension” glaucoma cases, which progress without chronic IOP elevation, might occur as a result of mechanical stresses in the optic nerve and retina, induced by either systemic conditions or medication- or lifestyle-induced IOP spikes.

Clearly, IOP significantly influences glaucoma progression and severity, but while a large clinical trial demonstrated that reduction of IOP is effective in slowing the disease in most patients, it also demonstrated that 45% of eyes treated with IOP lowering therapy showed significant disease progression over the 5-year study period [10]. Similar findings were obtained by others, suggesting that, while the disease in some patients progresses at a slow enough rate to allow them to retain vision throughout their lives, essentially all glaucoma patients are getting worse (reviewed in [11]).

Importantly, current glaucoma treatments, relying entirely upon reduction of IOP, become less effective as the disease progresses [12,13], suggesting that additional pathomechanisms increasingly participate at advanced disease stages. Patients with existing glaucomatous damage are at the highest risk for additional vision loss and frequently require very aggressive treatment [14]. Data from a number of studies have indicated that patients with higher mean deviation at standard automated perimetry at the onset of the trial are less likely to respond to treatment and are more likely to continue to exhibit vision loss than those with a lower mean deviation [14,15]. Finally, it appears that maximum IOP is often more predictive of future vision loss than average IOP, suggesting that an initiating event can predispose to subsequent damage [16].

Together, these clinical observations indicate that the pathophysiology leading to RGC death in glaucoma may change during the progression of the disease. Initial damage may largely be correlated to IOP, but eventually, secondary damage mechanisms that are not mitigated by lowering IOP become more established as the disease progresses. It is possible that the relative contributions of these secondary mechanisms to RGC damage eventually rival or overtake that of the initiating event(s), especially in patients that continue to experience vision loss despite maximal reduction of IOP. Progressive and irreversible structural changes of the optic nerve head (ONH) and the lamina cribrosa are characteristic of glaucoma [17], and it is likely that these changes increasingly compromise axonal survival in the ONH. However, there is also substantial experimental evidence demonstrating that immediate early innate immune responses, facilitated by multifactorial mechanical, hypoxic, or metabolic stresses [18,19,20,21,22], are succeeded by a sustained adaptive immune response and leucocyte translocation into glaucomatous retinas and optic nerves [23,24,25].

In this review, we will focus on the factors contributing to the innate immune responses occurring in the retina early in glaucoma and how these could lead to adaptive immune responses during later stages of the disease. These events have been divided into early, intermediate, and late events. Our use of these terms is not based on clinical parameters such as nerve fiber layer thinning or ONH cupping. Rather, these reflect the temporal sequence of molecular events in the retina. Furthermore, as a result of the asynchronous progression of retinal damage in glaucoma patients, some portions of the retina may undergo late events, while others experience early events or may even be unaffected at the same time. However, within a specific retinal region, late events will not occur unless early and intermediate events have taken place.

## 2. Early Events

### 2.1. Metabolic and Mitochondrial RGC Stress

Clinically glaucoma is defined as visual field loss associated with ONH changes. However, on a cellular level, the retina responds rapidly to stress and these early events can be observed prior to RGC loss. Owing to the high energy requirements of RGC, their survival, axonal transport, and electrophysiological functions are vulnerable to disruptions of their metabolic supply and dependent on functional mitochondria. This is particularly true for the portion of the RGC axons within the retina and ONH that are not myelinated as it requires more energy for the generation of action potentials [26]. Emerging data indicate that impaired RGC energy metabolism, owing either to decreased nutrient supply or mitochondrial dysfunction, is an early event in glaucoma etiology (reviewed by [27,28]. Damage to mitochondria may accumulate over many years and a functional decline could be caused by blue light-induced damage to mitochondrial proteins [29,30], or accumulating mitochondrial DNA damage [31]. Either of these events will lead to the production of reactive oxygen species by damaged mitochondria that further degrade their function [32].

A second cause for metabolic deprivation in RGC in some individuals could be impaired blood flow to the ONH and the retina due to faulty autoregulation to compensate for reduced retinal perfusion pressure resulting from increased IOP [33,34,35]. Retinal perfusion is difficult to quantitate, but insufficient perfusion is also likely to result in reduced oxygenation of the tissue. One expected consequence of hypoxic conditions is the accumulation of hypoxia inducible factor α (HIF-1a), which has been detected in mouse models of glaucoma [36,37] and in human glaucomatous retinae [38,39]. Additional support for a causative role of metabolic dysregulation for RGC damage in glaucoma is provided by animal studies demonstrating that increasing energy supply in mice mitigates glaucoma damage [40,41]. Similarly, a recent clinical study suggests that supplementation with nicotinamide, a precursor of NAD+, improves inner retinal function in glaucoma patients [42].

Mitochondria are known to play a central role in orchestrating both innate and adaptive immune responses. Consequently, the early loss of mitochondrial functionality in glaucoma not only directly affects axonal transport and increases oxidative stress in neurons, but also inevitably facilitates a neuroinflammatory cascade of events, including activation of microglia and astrocytes, and neutrophil attraction [43]. A new pathway of mitochondrial damage involving activation of innate immune complex, the inflammasome, and its downstream product gasderminD (GSDMD) has potential implications in glaucoma pathogenesis. Activation of the inflammasome and gasderminD maturation has been shown in acute models of ocular hypertension (OHT) [21,44], but their impact on RGC mitochondrial status in glaucoma remains to be elucidated.

### 2.2. Release of Extracellular ATP

One of the key metabolic disturbances of cells exposed to mechanical and ischemic stresses during IOP elevation is an increase of extracellular ATP and intracellular calcium. ATP release, apart from mechanical injuries, is implicated in numerous central nervous system (CNS) and retinal diseases and has been observed in primate and rodent models of glaucoma [45], including DBA2J retinas [46], and in human eyes with glaucoma [47]. During neuronal activity, ATP is released from the synaptic cleft and its extracellular accumulation is tightly controlled by the cell surface NTPDase1. However, under stress or injury, the efflux of ATP into the extracellular space occurs from either injured or dead cells or from viable glia and neurons via activated pannexin and connexin hemichannels [48,49].

Extracellular ATP is neurotoxic through activation of purinergic Panx1-P2X7 signaling, Ca^2+^ release from the endoplasmic reticulum, and the uptake of extracellular Ca^2+^ through activation of these channels [5,50,51]. In addition, however, ATP danger signaling is highly pro-inflammatory and facilitates neuroinflammation by attracting microglia to the inner retina and optic nerve and activating astrocytes [52,53,54]. Another consequence of ATP release is inflammasome activation and rapid cytokine production [18,44,55,56].

### 2.3. Inflammasome Formation and Signaling

Inflammasomes are cytosolic multiprotein complexes that become activated upon cellular infection or stress and cause the maturation of proinflammatory cytokines such IL-1β and IL-18. The assembly of the inflammasome complex containing NOD-like receptor proteins (NLRPs), apoptosis-associated speck-like protein containing a CARD (ASC), and caspase1/11 precursors requires two distinct signals: transcriptional “priming” via MyD88-NF-κB pathways (Signal-1) and complex assembly signaling via purinergic Panx1-P2(R)X7 signaling [55,57,58,59]. Cytokine maturation by inflammasomes requires activities of proinflammatory caspases-1 and -4 (caspase-11 in mice) that execute the cleavage-activation of the IL-1β and IL-18 precursors.

Inflammasomes were initially described in professional immune cells of the innate immune system, but it has become clear that neuroinflammation can also be initiated by inflammasome activation in CNS glia or neurons [60,61,62]. In the retina, activation of the inflammasome and rapid cytokine production has been demonstrated within hours after IOP elevation in both acute [18,44] and chronic glaucoma models [55,56].

A growing body of experimental evidence obtained in animal glaucoma models [19] and observed in human glaucomatous tissues [56,63] shows markers of inflammasome activation in affected retinas and optic nerves. The increased inflammasome activity in astrocytes is strongly associated with the key risk factors of glaucoma: IOP elevation [55], oxidative stress [64,65], and aging [66,67]. Moreover, signaling pathways that are implicated in glaucoma, specifically responses to mechanical and ischemic stress, are known to be involved in the regulation of the inflammasome: TNFα, TLR4 receptors, MyD88, and NF-κB are essential parts of the transcriptional priming of the complex [18,68,69,70,71]. Signaling via JNK [72,73], P2X7 receptors [74], TRPV1/4 channels [75,76], and Panx1 channels [18] is also mechanistically involved in the regulation of the inflammasome complex assembly.

The overlap in upstream regulators strongly suggests that the inflammasome might play a role as a downstream effector in OHT-induced degenerations, including glaucoma. Consistent with this role, genetic ablation or pharmacological inhibition of caspase-1; caspase-8; or inflammasome regulators, including Panx1, IL-1, and P2X receptors, protects retinal neurons in several injury models [18,58,77,78,79,80,81]. Therefore, the activation of the inflammasome is an early pathway pivotal to RGC dysfunction and neuroinflammation.

## 3. Intermediate Events

### 3.1. Neuroinflammation

Neuroinflammation is generally defined into immune responses to injuries and stressors relevant to the CNS. This response differs from inflammation in other systems where monocytes are the primary responders. In general, inflammatory responses to acute injury or stress are protective as they promote faster tissue repair. However, sustained neuroinflammation, which is typically associated with chronic stresses and aging, is neurotoxic. Neuroinflammation in glaucoma was historically considered to be the result of microglial and astrocytic responses to ocular hypertension, the onset of complex mechanical and metabolic stress, or neuronal injury death, and that it affects both the neural retina and the optic nerve. Indeed, transcriptomic profiling of microglial and astroglial cells has revealed differential activation of multiple pro-inflammatory pathways in these cell types [82,83]. However, recent reports challenged this paradigm as they showed two additional sources of potent pro-inflammatory responses: a localized perivascular infiltration of blood-borne monocytes [23,24,84,85] and induction of innate immune responses in the retinal ganglion cell layer [18,19,21,44,61]. In addition, Muller glia have also been suggested to contribute to neuroinflammation in glaucomatous tissue via dysregulation of metabolic control functions, cytokine release, and other pro-inflammatory responses.

Chronic elevation of IOP or repeated acute IOP spikes are known to cause neuroinflammatory activity in the retina. Metabolic disruption due to local hypoxia and mitochondrial dysfunction, a build-up in reactive oxygen species (ROS), damage-associated molecular patterns (DAMPS), and hemoglobin leakage resulting from vascular permeation or focal bleeding at the ONH are also able to initiate neuroinflammatory responses. Heat-shock proteins (HSP 27 and 70) and high mobility group protein 1 (HMGP1) are elevated in the glaucomatous retina and ONH [19,86,87] and are recognized by microglia via toll-like receptor 4 (TLR4), which signals NF-κB [19,88].

Through the induction of these innate immune pathways, the initial stresses trigger pathological changes in glial and infiltrating cells’ responses. These include increase in extracellular ATP; maturation of pro-inflammatory TNFα, IL-1, and IL-6 cytokines [52,57,75,89]; deposition of complement C1q on ganglion cell somas and dendrites [90,91]; and the release of other danger factors known to induce neuroinflammation, such as alarmin HMGB1 [19] in the retina and optic nerve. Recent studies, investigating cytokine release in glia-microglia and glia-neuron co-cultures, further demonstrated that microglial conditioning plays a key role in causing astroglial differentiation into the neurotoxic A1 type that is also capable of facilitating RGC injury [92,93].

Consistent with the proposed role of neuroinflammation in glaucoma, the blockade of different pathways of pro-inflammatory cytokine signaling resulted in RGC protection in animal models [70,75,94,95,96,97]. Pro-inflammatory cytokines, including the IL-1 family, are produced by glial, microglial, and neuronal cells in the retina in response to ischemic injury and in glaucoma [77,98,99,100,101]. Importantly, their production is controlled by inflammasome activation and significantly increases after exposure to elevated intraocular pressure [21,102].

A functional role for inflammation is firmly established in animal glaucoma models [103], but similar data in human glaucoma remain to be established. This is due in part to difficulties obtaining high quality human tissue with well described ophthalmologic findings, but also because the progression of the disease occurs over many months and typically does not affect all areas of the retina simultaneously. Finally, glaucoma patients represent a much broader spectrum of etiologies and cellular responses than inbred mice and typically receive glaucoma medications, making the interpretation of findings challenging. Consequently, most studies have characterized molecular changes associated with the onset and progression of glaucoma using comparative immunohistochemical methods. However, data have generally confirmed the findings on inflammatory pathway activation and microglial and astroglial activation in animal models. The analyses of primary optic nerve astrocytes, whole optic nerve, and retinal tissues showed evidence of neuroinflammation, including the pro-inflammatory signaling via TNF receptors, Casp1-, and NLRP-containing inflammasomes [52,56,83,104].

### 3.2. Complement Cascade Activation

**Figure 1:**
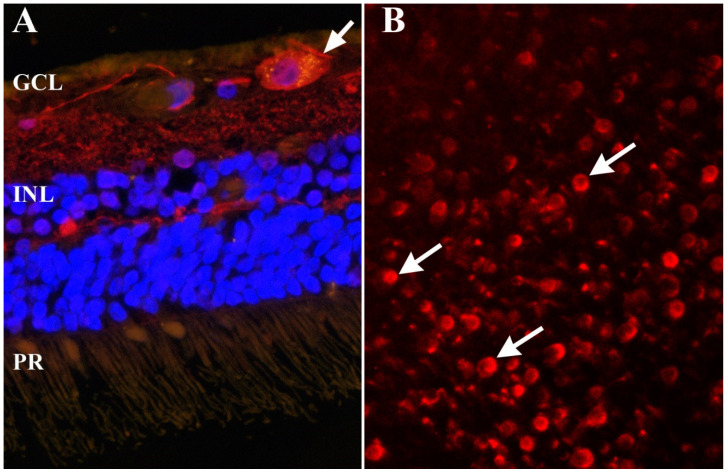
Formation of membrane attack complex (MAC) in the human retina. (**A**) Detection of the retinal ganglion cell (RGC) marker g-Synuclein (red) and MAC (green) in a sagittal section of a human glaucomatous retina. Deposition of MAC is apparent as yellow punctate labeling on the RGC (arrow) nuclei were labeled with DAPI (blue) to facilitate orientation. (**B**) Whole mounted retina of a second human eye with glaucoma. In some regions of this eye, MAC (red) could be detected in a large number of cells in the retinal ganglion cell layer (arrows). GCL: ganglion cell layer, INL: inner nuclear layer, PR: photoreceptor cells.

Involvement of the complement cascade in the pathobiology of RGC degeneration, and especially in glaucoma, has been the focus of numerous studies using animal models, human eye donor tissue, and aqueous humor (AH) samples from glaucoma patients. Altered expression and increased deposition of C1q and C3 on RGC have been demonstrated in human glaucomatous retinas, where upregulation of C1q seems to be correlated with disease progression [91,105,106]. More recent proteomic studies on AH composition derived from glaucoma patients further confirm these early observations [107]. The findings indicate that specific drug treatments alter the complement cascade [108], whereas levels of C4A, C4B, and C8B in AH are associated with abnormal visual field parameters [109]. Besides increased detection of the early components of the classical complement cascade (C1q, C1s, C1r, C3, C4a, C4b), some studies report accumulation of downstream complement compounds, such as C5, C6, C7, C8a, C8b, C8g, and C9, in glaucomatous retinas [91,110]. These downstream complement compounds are required to assemble the membrane attack complex (MAC), which subsequently can lead to lysis of targeted cells if MAC is not inhibited. MAC can be observed in association with RGC in the retina of glaucoma patients (Figure 1), but whether this leads to their demise has not been firmly established.

While activation of the complement system in glaucoma has been well established, the origin of specific complement components is the subject of ongoing investigations. Early studies revealed that the components of the classical complement cascade such as C1 and C3 are synthesized locally within the retina [91,111]. The presence of downstream complement proteins necessary for MAC formation (C5 through C9) has also been confirmed in the retina and AH of glaucoma patients, but their synthesis by retinal cells has not been demonstrated. However, the majority of complement proteins in the bloodstream are synthesized in the liver, and it is likely that the MAC components accumulating on RGC are derived from the blood stream and enter the retina as a result of increased permeability of the blood–retina barrier in glaucoma [112,113]. The activation of the complement cascade itself can lead to further vascular permeability through the generation of the anaphylatoxins C3a and C5a that facilitate blood–brain barrier breakdown in CNS disorders [114,115], and there are indications that this is also the case in glaucoma [116,117].

The notion of blood–retina barrier leakiness in glaucoma is further supported by proteomic comparison of antibody patterns in blood serum and AH obtained from healthy and glaucoma subjects. While significant differences between serum antibodies and AH antibody patterns are evident in healthy individuals, autoantibody compositions in serum and AH are almost identical in glaucoma patients [118]. This hypothesis might provide an explanation as to why retinal MAC formation is rarely observed under healthy conditions. While several reports have proposed that basal activation of the classical and alternative complement pathway in eye tissue significantly contributes to its immune privilege, there is also a body of evidence demonstrating the presence of regulatory complement compounds such as C1 inhibitor, complement factor H, vitronectin, and clusterin in glaucoma patients [110,119,120]. This suggests a balance between complement activation and inhibition that could prevent damage in healthy eyes, but may be shifted in glaucoma, leading to RGC damage. Intervention at the C3/C5 level for potential glaucoma therapies has been proposed and encouraging preclinical results show a significant decline, but not complete protection, of glaucoma progression in DBA2J mice [121,122,123]. Another study using cobra venom factor for complete complement cascade inhibition in rats after laser-induced OHT also reports RGC protection [124]. Thus, complement activation, including MAC formation, seems to contribute directly to RGC degeneration in glaucoma, but may also indirectly trigger other inflammatory events.

### 3.3. Inflammasomes Mediate Crosstalk between the Innate and Adaptive Immune Systems

Since the discovery of GsdmD-NT pores as major conduits of the secretion of the mature IL-1β and IL-18 cytokines [125,126], their pivotal role has been recognized in spreading danger signaling, neuroinflammation, and neurotoxicity in a number of CNS pathologies. Gasdermins have been implicated in vascular damage in hyperglycemia-induced diabetic retinopathy [127,128,129,130] and in neuronal loss in animal models of ischemic stroke, Alzheimer’s, age-related macular degeneration [131,132,133], and glaucoma [44,134]. Cleaved GsdmD-NT oligomerizes to form megapores that permeate membranes [135] to the mature IL-1β and IL-18 cytokines [125,126] and other soluble proteins, such as pro-inflammatory alarmin/HMGB1 [136]. These pores are formed in plasma and mitochondrial membranes and contribute to ionic disturbance, cellular microenvironment toxicity through the release of danger factors, and pro-apoptotic signaling through cytochrome C release from the mitochondria [137].

Combined, these factors serve as potent attractants and activators of microglia and peripheral macrophages, and subsequently, retinal macroglia. Notably, a key role of proinflammatory IL-1β cytokines in neurotoxic crosstalk between microglia and astrocytes has been demonstrated [92,93]. Consistent with a pivotal role in neuroinflammation, the inhibition of the glial NLRP3 inflammasome by MCC950 blocked the release of TNFα [138], a major cytokine implicated in glaucomatous pathology released primarily by activated glia. Likewise, the suppression of both glial NLRP3 and neuronal NLRP1 inflammasomes by the flavonol Kaempferol protects RGCs in an acute OHT injury model and is correlated with reduced activities of caspases 8 and 3 as well as decreased NF-kB, and JNK signaling [139].

Taken together, these findings demonstrate that the activation of GsdmD-NT pores mediates cytokine release that sustains and propagates proinflammatory signaling in the retina similar to a “cytokine storm” induced by sepsis or viral infection [140].

## 4. Late Events

### 4.1. Inflammasome Mediated RGC Death

It is widely accepted that neuronal death in glaucoma occurs via apoptosis [141,142] while the inflammasome causes pyroptosis, a subtype of regulated necrotic cell death dependent on caspases 1, 8, and 11 [143,144]. However, an increasing body of evidence links inflammasome also to apoptotic signaling [145]. A series of reports showed the pores formed by GsdmD and two other isoforms, GsdmA and GsdmE, can insert and permeate mitochondrial membranes and mediate the release of cytochrome C, a potent inducer of pro-apoptotic caspase-3 [146]. This discovery directly links proteolytic activation of gasdermins with apoptotic signaling and death [137]. It is therefore reasonable to assume that activation of mito-GsdmD pores in RGCs of ocular hypertensive retinas causes both early mitochondrial dysfunction and subsequent apoptotic death.

Cell death pathways involved in glaucoma include both intrinsic and surface receptor-mediated pro-apoptotic signaling. One of the first characterized and the most defined is signaling via TNF receptors 1/2 [52,147,148]. More recent studies have revealed the role of P2(R)X7 [48,74,149], TRPV1/4, Panx1 [75], TLR4 [21,150], and mTOR [151,152], pathways using animal models of retinal ischemia-reperfusion injury and induced glaucoma. The link between the intrinsic apoptotic cell death signaling pathways and activation of JNK and DLK kinases has been demonstrated in a number of studies using optic nerve crush models indicating a role in the death of RGCs [153,154,155,156]. It remains to be determined which of these pathways are critical in early versus advanced disease stages.

### 4.2. Autoimmune Responses

In inducible animal models of glaucoma, typically, only one eye is manipulated, whereas the contralateral eye remains unaltered. However, the fellow eye does not remain unaffected and, as early as 2005, studies have demonstrated that the normotensive fellow eye in a rat model of OHT also exhibits altered GFAP labeling patterns, similar to those observed in glaucomatous eyes [157]. These observations were subsequently confirmed and expanded upon by other investigators in both rat and mouse models who documented both macro- and microglial responses in the normotensive fellow eye [158,159] and eventually a reduction of RGC in the fellow eyes [84,91,94]. These findings suggest that mechanisms capable of degrading healthy RGC in eyes without elevated IOP become established as a result of glaucoma. Evidence also exists indicating that the fellow eye in patients with unilateral glaucoma is at higher than average risk of developing vision loss. Vision loss in the fellow eye has been observed in 30% of cases with unilateral normal tension glaucoma [160] and in 33% of cases of unilateral malignant glaucoma [161]. Other investigators have reported retinal nerve fiber layer defects in at least 33% of unilateral glaucoma cases [162] as well as in unilateral POAG [163], pseudoexfoliation glaucoma [164,165], and angle closure glaucoma [166]. Finally, 50% of cases with unilateral trauma induced angle recession glaucoma developed either frank glaucoma or suspicious visual fields in the fellow eye [167].

**Figure 2:**
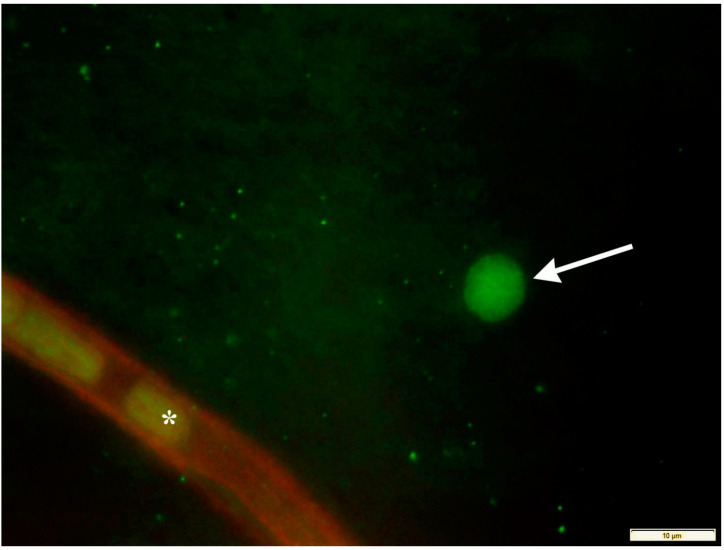
Detection of CD3 positive cells (green) by confocal microscopy in the whole mounted retina of a healthy human eye. While most cells reacting with an anti-CD3 polyclonal antibody (Abcam) are visible within the vasculature (asterisk), a small number of extravasated T-cells (arrow) are regularly observed.

Involvement of immune-dependent events in the pathology of glaucoma is one explanation for the observed effects on the contralateral eye and, although the retina is an immune privileged tissue, extravasated T-cells can be observed at a low frequency in nearly all human donor eyes (Figure 2). A role for T-cell mediated immune responses in glaucoma has been suspected for some time [168], and more recently, at least two laboratories have demonstrated that adoptive transfer of T-cells from glaucomatous mice causes RGC loss in normal recipients [23,25]. A role for adaptive immunity is further supported by a study demonstrating that RAG1^−/−^ mice lacking T- or B-cells lose significantly fewer RGC in eyes with elevated IOP than immune sufficient control animals [84]. The same study also demonstrated that normotensive fellow eyes sustained damage only in normal mice, but not in the absence of T- and B-cells. Together, these data strongly indicate that an immune response significantly contributes to RGC damage in glaucoma.

A mechanistic explanation of how an immune response is initiated in glaucoma has not been firmly established, but some data indicate that it is a recall of immune memory rather than a de novo induction to a glaucoma specific antigen. Data have been presented suggesting that immune responses in glaucoma could be the result of activation of T-cell response directed against heat shock proteins (HSPs) [23]. HSPs are a family of chaperones that fulfill roles in protein folding and degradation. Several HSPs are rapidly upregulated in response to cell stress and are then displayed on the cell surface [169]. This is also the case in glaucoma and increased expression of HSP60 and HSP27 has been demonstrated in the retina and ONH of humans with the disease as well as in monkey and mouse models [23,87,170]. HSPs are among the most highly conserved proteins in phylogeny and the amino acid sequences of human HSPs often differ from those of bacteria by less than 50%. It is conceivable that individuals develop immune responses against HSP of, e.g., gut bacteria, at some point of their lives, and indeed antibodies directed against HSP60 are frequently detected in glaucoma patients [171]. Whether or not autoantibodies contribute to glaucomatous damage in humans remains a matter of debate, but it has also been demonstrated that POAG patients exhibit a more robust T-cell response upon stimulation with HSP 60 or HSP 27 than controls [23,172]. Thus, it is conceivable that elevated expression of HSP results in increased loading of their proteolytic fragments on major histocompatibility complex I and II, which is also expressed at elevated levels in the glaucomatous retina, that can then serve to initiate T-cell recall responses [22,173].

Alternatively, it is possible that an RGC-directed immune response may be the result of loss of tolerance in some individuals. Multiple mechanisms exist to induce immunologic self-tolerance to maintain autoimmunity. However, these are frequently imperfect and some reports suggest that up to one-third of CD4+ T-cells may be autoreactive, but are restrained by T-regulatory (Treg) cells [174]. This indicates that autoimmune responses can result from insufficient tolerance mechanisms and do not necessarily require a specific priming event. Loss of tolerance can result from a variety of causes that are associated with glaucoma, including aging and persistent inflammation, but it is often the result of insufficient Treg activity. Whether or not this is the case in patients remains to be determined, but studies have been published suggesting that the Treg population in POAG donors differs from that of controls [172,175].

One critical factor in both scenarios is the presence of a pro-inflammatory milieu in the retina and ONH that degrades the immune privileged environment and results from the neuroinflammation events described above. Increased levels of the cytokines TNFα and IL-1 β have been demonstrated in the glaucomatous retina [63,176]. TNFα in particular promotes diapedesis and is a potent stimulator of effector T-cells, while simultaneously inhibiting inflammation reducing Treg cells [177]. Death of RGC may then occur either as a result of further aggravation of retinal glial responses or through direct T-cell mediated cytotoxic mechanisms that induce RGC death. These could include fas/fas ligand mediated apoptosis of RGC [178]. While pro-inflammatory signaling most likely originates in the retina and ONH, it is intriguing to note that systemic inflammation states have been shown to contribute to chronic neurodegenerative diseases and a report linking oral inflammation with POAG disease progression has been published [179,180].

Firm evidence that adaptive immune responses contribute to vision loss in human patients is yet to be presented. However, if immunity does indeed contribute to vision loss in patients, a number of novel therapeutic approaches may be added to current therapy. For example, Etanercept, a TNFα inhibitor widely used to control chronic inflammatory diseases such as rheumatoid arthritis, prevents approximately 50% of RGC loss in a rat model of glaucoma [181]. Furthermore, patients using medications with anti-TNF properties experienced significant protection from glaucomatous damage in a large retrospective study [182]. An alternative approach may be the use of immune modulatory treatments that boost Treg cell activity in order to regulate or suppress immune responses. Studies in rats [183] and subsequently in mice [152] have shown that treatment with rapamycin offers significant protection of RGC in glaucoma models. Rapamycin, or sirolimus, is an FDA-approved immunosuppressant that generally enables a tolerogenic state by inhibiting proliferation of T, B, and NK effector cells and conversely stimulates Treg cell proliferation and activity [184]. Treg cells may then be recruited from the blood stream to further suppress the activity of T effector cells or may even be generated in the retina [185]. An alternative approach may be to inhibit the activity of fas signaling. Studies have indicated that both synthetic fas receptor antagonists as well as retinal overexpression of a decoy fas ligand provide substantial improvements in RGC survival in mouse models [186,187].

## 5. Conclusions

**Figure 3:**
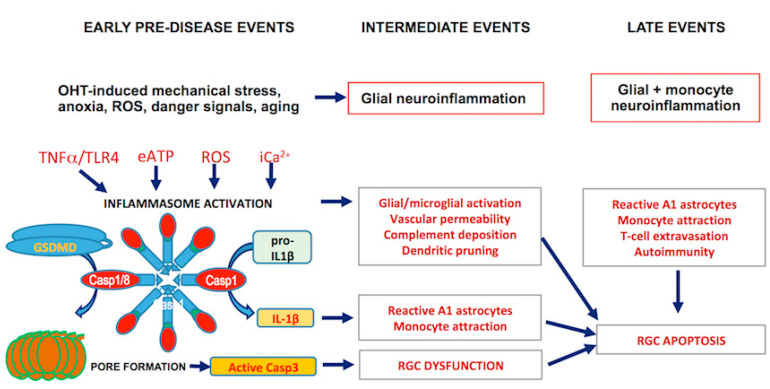
Schematic representation of the temporal sequence of molecular events during the development and progression of glaucomatous damage in the retina.

Taken together, a picture emerges of the trajectory of RGC damage in glaucoma (Figure 3). In this scenario, RGC stress leads to quick alarm signaling that induces glial responses, leading to inflammasome formation and the production of cytokines. Chronic neuroinflammation compromises retinal immune privilege, degrades the blood–retina barrier, and attracts T-cells. These extravasate into the proinflammatory environment of the glaucomatous retina and may, in some individuals, cause further damage to RGC. Although some aspects of this proposed sequence of events remain to be determined, it is encouraging that immunomodulatory therapies have significantly advanced during the past decade and could be used in addition to IOP lowering drugs to stop the progression of vision loss in some glaucoma patients.

## Data Availability

No data were generated for this review article.
